# Efficacy of fluralaner solution administered to egg layer chickens through drinking water for control of northern fowl mite (*Ornithonyssus sylviarum*)

**DOI:** 10.1186/s13071-025-07240-w

**Published:** 2026-01-21

**Authors:** Alec C. Gerry, Bradley A. Mullens, Levi Zahn, Faris Jirjis, Amy C. Murillo, Caleb B. Hubbard, Zikun Wang

**Affiliations:** 1 Department of Entomology, University of California, Riverside, CA 92521 USA; 2 Intervet Inc. (Merck Animal Health), Rahway, NJ 07065 USA; 3 Department of Entomology, Plant Pathology & Weed Science, New Mexico State University, Las Cruces, NM 88003 USA

**Keywords:** Poultry, Mite, Pest management, Ectoparasite, Oral, Veterinary

## Abstract

**Background:**

The northern fowl mite (NFM), *Ornithonyssus sylviarum*, is one of the most important external parasites of commercial poultry in the USA. NFM feeds on blood, causing irritation and stress to infested birds and potentially reducing egg production in flocks with high levels of mite infestation. Fluralaner is a systemically active insecticide and acaricide. We report on two studies that evaluated the efficacy of fluralaner administered to layer chickens in medicated drinking water through two single doses of 0.5 mg fluralaner per kg chicken body weight at 7 days apart for control of NFM.

**Methods:**

In two separate studies, white Leghorn chickens (*Gallus gallus domesticus*) were exposed to NFM so that they developed mite infestations. The first study was a dose confirmation study (*n* = 64 pullet birds per treatment group). The second study was a field efficacy study (*n* = 400 layer birds per treatment group). Once infested with NFM, birds were assigned to Medicated or Control treatment groups. In the Medicated group, a fluralaner solution was administered through medicated drinking water on study day 0 and again on day 7. The Control group received only unmedicated drinking water. NFM present in the vent region of birds were recorded prior to treatment (day −7 for dose confirmation and day −5 for field efficacy studies) and post-treatment on days 2, 8, 14, 19, and 28. In each study, product efficacy was determined by comparison of mite counts on Medicated and Control birds.

**Results:**

The number of mites was significantly reduced on Medicated group birds relative to Control group birds by day 2. At day 2 post-treatment, 99% control efficacy (> 99% for geometric mean) was achieved in the dose confirmation study and > 96% (> 99% for geometric mean) control efficacy was achieved in the field efficacy trial. Control efficacy in both studies exceeded 99% from day 8 to day 28. There were no adverse health impacts observed in birds treated with fluralaner.

**Conclusions:**

This study confirms the effectiveness of fluralaner for control of NFM when administered to chickens through drinking water as two single doses of 0.5 mg/kg chicken body weight at 7 days apart.

**Graphical Abstract:**

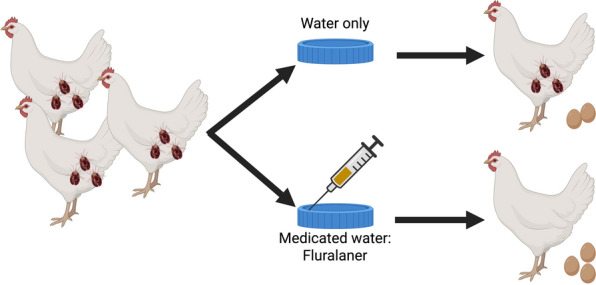

## Background

The northern fowl mite (NFM), *Ornithonyssus sylviarum* (Canestrini and Fanzago, 1877), is one of the most common and important external parasites (ectoparasites) of domestic chickens (*Gallus gallus domesticus* L.) and other commercial poultry in the USA [1, 2]. Northern fowl mite is also common on wild birds [3], providing a mechanism for the movement of these mites among commercial poultry facilities [4]. The NFM generally remains on the host during all life stages [5], though NFM also readily disperses among birds within a flock when mite abundance on individual birds is high (> 100 NFM) [6]. NFM can survive off the host for up to 35 days when the temperature is cool and humidity is high [7], allowing mites to survive cleanout periods between flocks in commercial poultry production and supporting rapid infestation of a newly introduced flock.

The NFM life cycle comprises five life stages (egg, larva, protonymph, deutonymph, and adult), with mites requiring a bloodmeal during the protonymph and adult life stages, while remaining life stages are nonfeeding [5]. Within 68–89 h of adult mites taking a bloodmeal, females deposit 1–5 eggs onto their avian host, and the resulting larvae emerge and develop to the first blood-feeding life stage (protonymph) [5]. The entire life cycle of NFM is quite rapid, requiring as few as 5 days from egg to adult [5]. Due to their rapid life cycle, NFM can quickly reach very high numbers on infested birds, often resulting in substantial irritation and potentially reducing feed conversion efficiency and egg production [4, 8–11]. On domestic egg-laying hens (“layers”), the vast majority of NFM congregate in the vent region (near the cloacal opening) [2]. Heavily infested birds often show discoloration and darkening of the feathers in this region due to accumulation of mite feces [2].

Fluralaner (carbamoyl-benzamide-phenyl-isoxazoline) is an isoxazoline compound that inhibits the γ-aminobutyric acid (GABA) and L-glutamate chloride channels in the central nervous system of arthropods [13–15]. A variety of formulations of fluralaner are registered for use in multiple veterinary species with numerous ectoparasite claims in many countries. Registered uses and formulations include systemic treatments for fleas, ticks, and mites on dogs and cats; for ticks on cattle; for lice in sheep; and for mites on chickens. Fluralaner administered to layers by oral gavage as two single doses of 0.25–1.0 mg/kg chicken body weight (BW) applied at 7 days apart provided > 90% control of NFM for up to 22 days even when treated birds were comingled with untreated, mite-infested birds [16]. In a small-scale trial to control another important poultry ectoparasite, poultry red mite (*Dermanyssus gallinae* [De Geer]), a single fluralaner dose administered orally to chickens through drinking water at 0.5–2 mg/kg BW gave 100% control of mites for up to 5 days post-treatment [17]. In a speed-of-kill study, 97% of poultry red mites were killed within 4 h of feeding on hens treated with fluralaner by oral gavage at 0.5 mg/kg BW within the previous 12 days [18]. Fluralaner also appears to exhibit contact toxicity to both NFM and poultry red mite [19, 20], though contact toxicity is low relative to oral toxicity [20].

In large-scale studies conducted in Europe to control poultry red mite, fluralaner administered orally in drinking water as two single doses of 0.5 mg/kg BW applied 7-days apart to poultry provided 100% control for up to 6 weeks in an aviary, > 99% control at 6 weeks on a free-range farm [21], and > 90% control for at least 8 weeks in a layer farm [22]. Similarly, fluralaner administered to layers in drinking water at two single doses of 0.5 mg/kg chicken BW applied 7 days apart provided > 95% control of a newly described feather mite *Allopsoroptoides galli* n. sp. (Analgoidea: Psoroptoididae) [23] for up to 70 days post-treatment and notably also reduced the appearance of skin lesions on treated poultry over this period [24]. However, fluralaner efficacy against NFM cannot be inferred from studies on other mite species due to differences in host-association, life history, and perhaps acaricide susceptibility among the mite species. Large scale trials of fluralaner efficacy against NFM are therefore warranted.

Administering fluralaner at a 7-day interval ensures that mites in a nonfeeding life stage when the first dose is administered will be exposed to an effective concentration of fluralaner during the next blood-feeding stage. This treatment strategy also extends treatment efficacy beyond the time gap between blood feeding by an adult female mite and blood feeding by its offspring during the protonymph stage (6 days for poultry red mite and 68–89 h for NFM) [5]. No treatment-related adverse events have been noted for breeder or layer chickens administered fluralaner at a dose equal to or even somewhat higher than 0.5 mg/kg BW [25, 26].

The current studies evaluate the efficacy and safety of fluralaner oral solution (10 mg/mL) for control of NFM when administered to egg-layer chickens (white Leghorn breed) through drinking water in two single doses of 0.5 mg/kg chicken BW administered 7 days apart.

## Methods

### Fluralaner oral solution

Fluralaner oral solution (10 mg/mL) was acquired from Merck Animal Health (Rahway, NJ, USA) and mixed into drinking water for oral consumption by chickens. The medicated drinking water was administered as two single doses of 0.5 mg fluralaner/kg chicken body weight (BW) 7 days apart. These studies were performed in compliance with protocols reviewed and approved by the Institutional Animal Care and Use Committee at the University of California at Riverside (UCR).

### Northern fowl mite

A research colony of NFM is maintained on chickens held in suspended wire cages within a small poultry house dedicated to this purpose at the University of California at Riverside (UCR) Agricultural Operations Poultry Research Facility. Because these “mite-maintenance birds” often support only low numbers of mites due to bird immune response following prolonged mite exposure [27], young chickens with no previous exposure to mites were acquired and comingled with the mite-maintenance birds, resulting in high mite numbers (≥ 1000 mites/bird) on the young “mite-amplifier” chickens within a few weeks’ time.

For this study, NFM were removed from mite-amplifier birds by aspiration of mites from the chicken body into glass pipettes containing a cotton plug to retain mites within the pipette. When each pipette was full (up to several thousand mites) the narrow end of the glass pipette was stoppered with clay and the pipette was retained on wet ice for no more than 3 h until mites were transferred to naïve chicken hosts.

### Mite count process

NFM were counted on birds by visual examination of the area near the vent (cloacal opening) of each bird, as the vast majority of mites congregate in this region on domestic egg-laying hens [12]. Mite counts were performed by holding the bird with the vent region facing up and away from the examiner under adequate lighting provided by a headlamp worn by the examiner. The feathers in the 4 × 6 cm area anterior to the vent were parted to expose the skin, and individual live mites on the skin and feathers in this area were rapidly counted and recorded. When mites were clustered in the vent region, the number of mites in each cluster was approximated and added to get an overall mite count. Visual examination required 1–2 min per bird and was performed by the same experienced researchers throughout each study.

### Dose confirmation study

Young (8 weeks old), beak-trimmed, vaccinated (Marek’s, NC/IB, Coccivac) white Leghorn chickens (“pullets”) (Hy-Line W-36 strain) (*n* = 160) were acquired from a commercial breeder and transported to the UCR Agricultural Operations Poultry Research Facility. Chickens were examined upon arrival at UCR and confirmed to be free of mites and other ectoparasites before being moved to a single open-sided, small-scale experimental poultry house where they were arbitrarily placed into 0.25 m^2^ wire cages at a density of four birds per cage. Neither the poultry house nor the cages within the house had been used to house chickens during the previous 2 years. Each cage contained a plastic water cup and an internal feed trough, with food (commercial pullet feed) and water replenished ad libitum daily. Cages were fitted with a metal litter tray beneath the cage to capture feces and spilled feed from the cage above, allowing cages to be stacked without feces falling to the cage below. Litter trays were cleaned weekly. Birds were exposed to local environmental conditions except that artificial lighting was provided to maintain a 14 h day length throughout the study. Duplicate numbered metal leg bands were placed on each chicken so that they could be identified individually.

Following a brief acclimation period of 1–2 h within the cages, chickens were infested with NFM by removing the cotton plug from a glass pipette containing NFM and tapping approximately 200 mites onto a white index card placed onto the floor of each chicken cage, thereby allowing mites to naturally locate a chicken within the cage. This method of mite infestation simulated a natural infestation process as mites locate and infest a new host. Chickens were subsequently examined every 2–3 days until a predetermined mite infestation target of ≥ 25 mites per individual bird was reached. This infestation target was reached at 5 weeks after NFM were introduced to birds, whereupon the study was initiated by first conducting a health examination of all birds (on day −8; Table 1) to exclude any birds that were ill or injured or had poor feather condition (no birds were excluded at this time).

**Table 1:** Activity performed by day in the dose confirmation study

Activity performed	Study day
Clinical examination of birds	−8
Pretreatment mite counts	−7
Randomized placement of birds into cages	−6
Measurement of drinking water consumption	−4, −3
Measurement of chicken body weights	−1, 6
Administered medicated or untreated drinking water	0, 7
Post-treatment mite counts	2, 8, 14, 19, 28

**Fig. 1:**
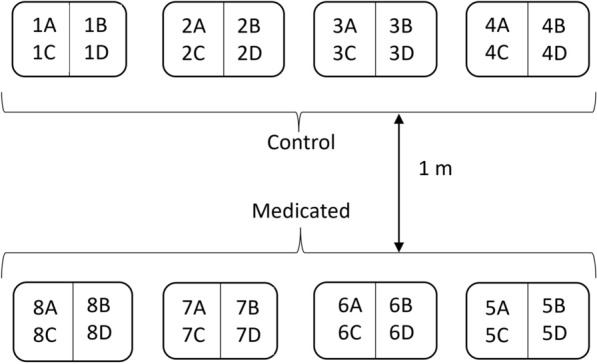
Facility design for the dose confirmation study. Cages separated by treatment group (Control or Medicated) to reduce possible cross-contamination, and each treatment group organized into four cage blocks with four cages in each block (Control: 1A–4D; Medicated: 5A–8D). Cages **A** and **B** were positioned above cages **C** and **D** in each cage block, with top and bottom cages separated by a metal shelf. Each cage held four birds with NFM counts on birds balanced across treatment groups, cage blocks, and cages within a block. Care was taken throughout the study to prevent transfer of fluralaner medication or mites among birds in different cage blocks and treatment groups

A pretreatment count of NFM on each chicken was recorded on day −7, and 128 healthy birds, all with pretreatment mite counts > 130, were selected for the study and ranked in pairs from highest to lowest NFM count. The following day (day −6), birds in each of the 64 ranked pairs were randomly assigned to each of the two treatment groups (Medicated or Control) to balance NFM counts among the two treatment groups. Cages for each treatment group were placed on opposite sides of the small-scale experimental poultry house, separated by 1 m of bare earth floor to restrict movement of NFM between the two treatment groups. Within a treatment group, cages were arranged into four separate cage blocks (Control: 1–4; Medicated: 5–8), with each cage block containing four stacked cages (labeled A–D) for a total of 16 cages per treatment group. Each cage was therefore given an alphanumeric code (1A–4D for the Control group and 5A–8D for the Medicated group) and each cage housed four birds (Fig. 1).

To balance NFM counts on birds among the four cage blocks within each treatment group, the 64 birds in each treatment group were ranked from highest to lowest NFM count, then sorted according to rank order into subgroups of four birds each to create 16 rank-order (high to low NFM count) subgroups per treatment group, with birds in each rank-order subgroup subsequently assigned randomly to one of the four cage blocks in the treatment group. Each cage block thus received birds with similar NFM counts. The 16 birds assigned to each cage block were similarly ranked by NFM count and further separated into four rank-order subgroups of four birds each, with birds in each rank-order subgroup randomly assigned to one of the four cages within the cage block. This ensured that NFM counts were similar across cages within a cage block and across cage blocks within a treatment group, as well as between the two treatment groups. The experimental unit was the mean mite count per cage for nonparametric analysis of treatment effect on each study day.

Fluralaner medicated drinking water or untreated drinking water was provided to the appropriate treatment group on day 0 (first dose) and again on day 7 (second dose). To ensure that each bird within a Medicated treatment group cage received the intended fluralaner treatment dose of 0.5 mg/kg chicken BW administered in drinking water over a 24-h period, daily water consumption and total BW for all four birds in each cage was determined. Daily water consumption for each cage of birds was measured over two consecutive days (days −4 and −3) by recording the initial and final volume of water (by weight) in the water cup each day, with daily water consumption for all birds within the cage calculated as the mean change in water volume over the 2 days. On the day immediately prior to administration of medicated drinking water to chickens, the BW of each chicken was measured and summed for all four birds in each cage to give a total chicken BW for each cage.

Medicated drinking water was prepared on the days of treatment (days 0 and 7) by diluting the 10 mg/mL fluralaner oral solution in water to make a 1 mg/mL stock solution. The volume of the stock solution added to the drinking water cup in each cage was calculated using the total BW for all chickens in the cage, with an additional 10% fluralaner adjustment added to the calculated value to compensate for waste/spillage of drinking water by the birds:

$$Vol stock solution \left(mL\right)=Total bird cage weight \left(kg\right)\times \frac{0.5 mg fluralaner}{kg}\times \frac{1 ml solution }{1 mg fluralaner}\times 1.1 overage$$

The calculated volume of the fluralaner stock solution was then added to drinking water to give a total volume of medicated drinking water in the water cup equal to 80% of the daily water consumption by all four birds in the cage. This ensured that birds within each cage consumed all medicated water within 24 h. Control treatment cages received a volume of untreated drinking water equal to 80% of daily water consumption. All cages received fresh untreated water as soon as their water cup was empty following treatment, after which water was again provided ad libitum for the remainder of the study.

On each treatment day, a 20 mL aliquot was taken from the fluralaner stock solution and also from untreated drinking water. Aliquots were labeled and stored in a laboratory refrigerator until transfer to the Merck Animal Health (Lawrence, KS, USA) analytical laboratory to determine fluralaner concentration by ultra performance liquid chromatography (UPLC) using an Acquity BEH C18 column (2.1 × 100 mm, 1.7 µm particle size [SN:186,002,352; Waters Corp, Milford, MA]). The injection volume was 7 µL, and the mobile phase was delivered at a flow rate of 0.5 mL/min. The column temperature was maintained at 50 °C, and analytes were detected by ultraviolet (UV) absorbance at 265 nm. The method provided a limit of quantitation (LOQ) of 0.1 µg/mL and a limit of detection (LOD) of 0.05 µg/mL for fluralaner. From these liquid chromatography (LC) assays, we confirmed that control birds were not exposed to fluralaner in drinking water, and the fluralaner dose administered to medicated birds was 0.47 mg/kg on day 0 and 0.54 mg/kg on day 7, with an average dose over both treatment days of 0.51 mg/kg.

### Field efficacy trial

**Fig. 2:**
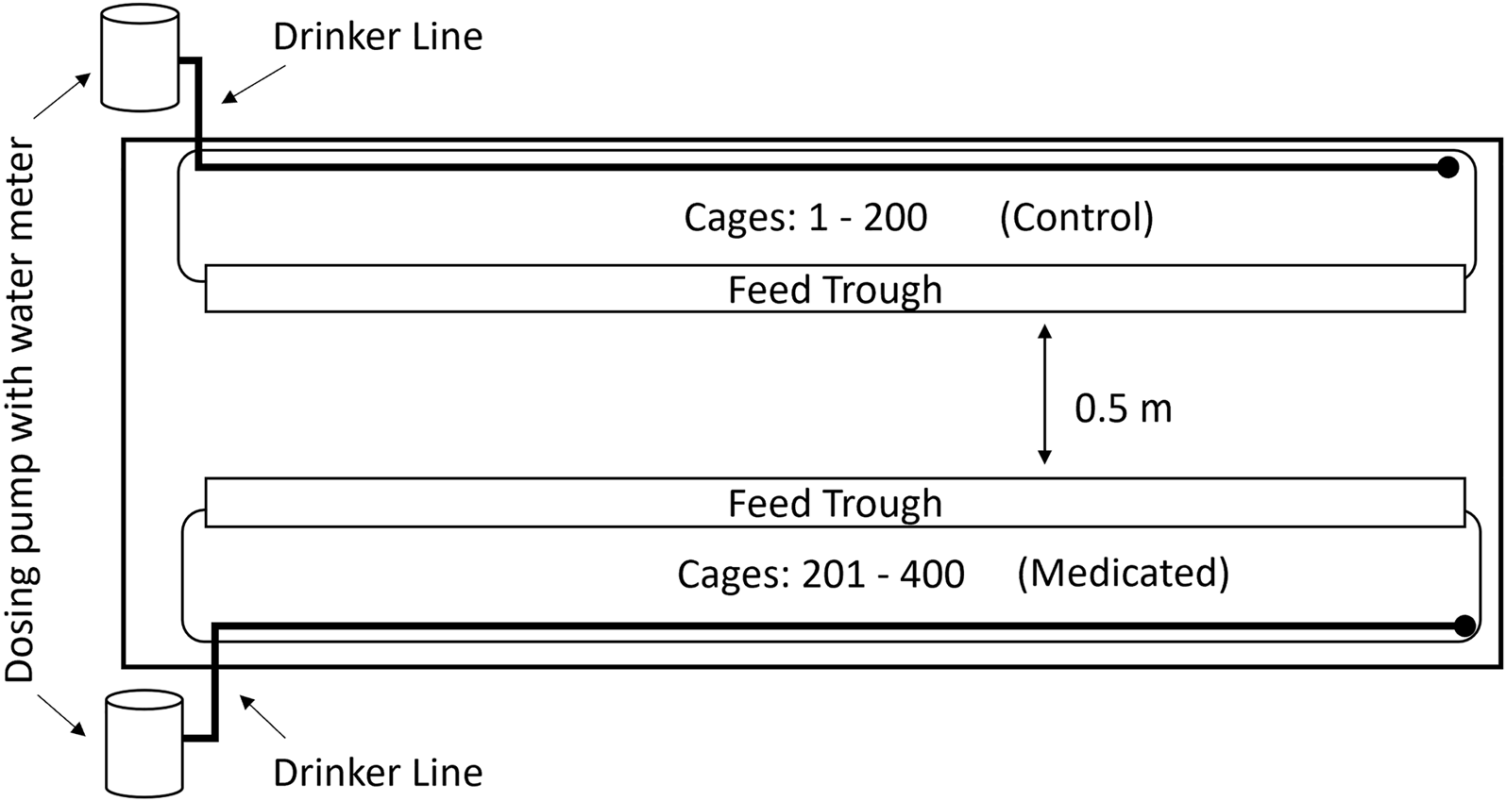
Facility design for the field efficacy trial. Cages separated according to treatment group (Control: 1–200; Medicated: 201–400) with two birds in each cage. Care was taken throughout the study to prevent transfer of fluralaner medication or mites between treatment groups, though poultry houses on this farm were open-sided (no walls) allowing access by wild birds, which were noted to occasionally rest on cages or feed troughs

Young (8 weeks old), beak-trimmed, vaccinated (Marek’s, NC/IB, Coccivac) white Leghorn chickens (Hy-Line W-36 strain) (*n* = 825) were acquired from a commercial breeder and transported to a commercial egg-layer farm in southern California. Chickens were examined upon arrival at the commercial farm and confirmed to be free of mites and other ectoparasites. Pairs of birds were arbitrarily placed into 1 of 400 suspended wire cages (0.58 m^2^) comprising two cage rows (200 cages per row) in an open-sided “California style” commercial poultry house (Fig. 2). Duplicate numbered metal leg bands were placed on each chicken so that they could be identified individually. Wire cages were numbered 1–200 and 201–400 in the two cage rows, respectively. The remaining 25 birds were placed into additional non-numbered cages of similar size at the far end of each cage row with no more than two birds per cage for replacement of study birds in the event of a bird health issue prior to the start of the study but were otherwise treated similarly to all study birds. Birds were exposed to local environmental conditions except that artificial lighting maintained a 16 h day length throughout the study.

A water “drinker” line and external feed trough ran the length of each cage row, providing birds with continuous access to water and food replenished ad libitum daily per standard commercial practice. Birds were provided a complete feed (commercial lay mash blend) free of antibiotics, growth promoters, or any other medications. Each cage was provisioned with nipple water drinkers drawing water from the shared drinker line for each cage row. A water meter and dosing pump installed at the start of each drinker line allowed for measurement of water consumption by all birds in a cage row and for controlled injection of solutions into the drinker line from an attached medication tank. Each drinker line also had a shut-off valve to prevent water flow and a removable end cap allowing the line to be emptied or flushed.

Birds were held under standard conditions at the commercial farm until they reached 16 weeks of age, at which time birds were infested with NFM by removing the cotton plug from a glass pipette containing NFM and tapping 50–100 mites from the glass pipette onto the base of the feathers surrounding the vent of each bird. Mites were given a few seconds to attach to the skin surface or feathers, before the newly infested bird was returned to her cage. All birds thus received a similar number of mites on the same date. This mite infestation process was used for the field efficacy trial so that initial mite numbers on all birds were similar with the intent being to reduce variation in mite numbers among birds. Birds were subsequently examined each week, and mites were counted (as described above) on one arbitrarily selected bird from each of 80 randomly selected cages within each cage row until a predetermined mite infestation target of ≥ 25 mites per individual bird was reached on a least 90% of birds examined. This infestation target was reached 3 weeks after NFM were introduced to the birds, whereupon the study was initiated the following week by first conducting a health examination of all birds (on day −6; Table 2) to exclude from the study any birds that were ill or injured or had poor feather condition. Excluded birds within the numbered study cages in each cage row were replaced using healthy birds from the replacement pool. A pretreatment mite count was performed for all birds on day −5.

**Table 2:** Activity performed by day in the field efficacy trial

Activity performed	Study day
Clinical examination of birds	−6
Pretreatment mite counts	−5
Measurement of drinking water consumption	−4, −3
Measurement of chicken body weights	−1, 6
Random assignment of cage row to treatment group	0
Administered medicated or nonmedicated drinking water	0, 7
Post-treatment mite counts	2, 8, 14, 19, 28

On day 0, the two cage rows were randomly assigned to a treatment group (Medicated or Control) (Fig. 2) immediately prior to the first administration of fluralaner solution to the Medicated treatment group. Fluralaner medicated drinking water or untreated drinking water was provided to the appropriate treatment group on day 0 (first dose) and again on day 7 (second dose). To ensure that each bird within the Medicated group received the intended fluralaner treatment dose of 0.5 mg/kg chicken BW administered in drinking water over a 6 h morning period, water consumption by all birds within each cage row was measured over a 6 h morning period on two consecutive days (days −4 and −3) using the water meter on each drinker line. Water consumption was calculated as the mean water volume delivered to the drinker line over the two measured periods. On the day immediately prior to administration of medicated drinking water to chickens, the total BW for all birds within a cage row was estimated by measuring the BW of 8 birds randomly selected from each 1/3 linear portion of each cage row (total of 24 birds per cage row) and then multiplying the mean BW of this subsample by the total number of birds within the cage row (total bird cage row weight).

Shortly after dawn on each treatment day, drinker lines for both cage rows (Medicated and Control) were drained by shutting off water flow and then removing line end caps. End caps were replaced after drinker lines were empty. The volume of the fluralaner oral solution added to the Medicated group drinker line was calculated using the total BW for all chickens in the cage row:

$$Vol fluralaner oral solution \left(mL\right)=Total bird cage row weight \left(kg\right)\times \frac{0.5 mg fluralaner}{kg}\times \frac{1 ml solution}{10 mg fluralaner}$$

This calculated volume of fluralaner oral solution was mixed into drinking water within a medication tank (stoppered glass Erlenmeyer flask) to give a total medicated drinking water volume (mL) = dosing pump injection rate (29.6 mL medicated solution/3.8 L drinking water) multiplied by the mean 6-h water consumption for the Medicated cage row. This resulted in an expected fluralaner concentration of 1.15 mg/mL in the drinker line of the Medicated treatment group. Control birds received only untreated drinking water through the dosing pump leading to the Control cage row. When the medication tank was emptied, 100 mL of untreated drinking water was added to the medication tank to rinse any remaining medicated drinking water into the drinker line. When the medication tank was again empty, drinker lines were flushed with 3.8 L untreated water per 10 m of drinker line.

On each treatment date, a 20 mL aliquot was taken from each medication tank immediately after preparation of the medicated drinking water. Medicated drinking water samples were labeled and stored in a laboratory refrigerator until transfer to the Merck Animal Health (Rahway, NJ, USA) analytical laboratory to determine fluralaner concentration using liquid chromatography. The actual fluralaner dose administered to birds was 0.51 mg/kg on day 0 and 0.46 mg/kg on day 7, with an average dose over both treatment days of 0.49 mg/kg.

### Mite counts and bird observations

During both studies, all birds were observed daily throughout the study to assess their general health, and mite counts were performed on days 2, 8, 14, 19, and 28. Mites were counted on all birds during the dose confirmation study or on a randomly selected representative subsample (*n* = 80) of the 400 birds in each treatment group during the field efficacy study. Study personnel conducting health observations and mite counts were masked as to which group of birds had received the fluralaner medication and which had received only untreated water. To avoid accidental transfer of fluralaner among treatment groups, all supplies and equipment used for the two treatment groups were kept separate, and study personnel wore disposable gowns and disposable gloves that were replaced after handling birds in each treatment group.

### Egg production during the field efficacy trial

Eggs were collected daily from all birds combined in each cage row (treatment group) from day −4 through the end of the trial. For each treatment group, daily egg production was calculated as the total number of eggs collected on that day divided by the number of birds in that treatment group. Eggs from both the Medicated and Control groups were subsequently destroyed and discarded.

### Statistical analyses

For both studies, initial analyses to evaluate the effect of treatment and trial day were performed using a general linear mixed model (PROC GLIMMIX) with negative binomial distribution and a maximum likelihood approximation (method = laplace). The negative binomial distribution was selected due to over-dispersion of mite count data. For the dose confirmation study, the cage was included as a random effect because four birds occupied the same cage. The nonparametric Kruskal–Wallis test (PROC NPAR1WAY) was used to further examine differences in the mean mite count of the four birds in each cage (dose confirmation study) and the differences in mite counts (field efficacy study) between treatment groups on each study day to address the skewed distributions of the treatment groups due to most (or all) treated birds having no mites. For both studies, differences in mite counts among sequential study days within each treatment group were also analyzed using the PROC NPAR1WAY procedure to evaluate changes in mite abundance by study day within a treatment group. Unless otherwise specified, mite count data are expressed as the mean number of mites ± standard error (SE).

Control efficacy was calculated for each day that mite counts were performed as:

$$Control Efficacy ( \%)=100\times \frac{{M}_{c}-{M}_{t}}{{M}_{c}}$$

where M_c_ is the mean mite count for birds in the Control group and M_t_ is the mean mite count for the Medicated (treated) group. Control efficacy was calculated using both arithmetic means and geometric means for mite counts. Geometric means were calculated due to the large fluctuation in mite counts across study days and to reduce the effect of outliers during the post-treatment period when Medicated birds were largely free of mites. Fluralaner treatment was considered effective at ≥ 90% control efficacy (arithmetic mean).

Differences in mean daily egg production among the two treatment groups were evaluated by paired *t*-test with mean egg production paired by study day. Mean daily egg production was visualized by a Q–Q plot indicating residuals were approximately normally distributed. All statistical analyses were conducted using two-tailed tests, with statistical significance defined as *P* ≤ 0.05. All analyses were performed using SAS v. 9.4 (SAS Institute Inc., Cary, NC).

## Results

### Dose confirmation study

**Fig. 3:**
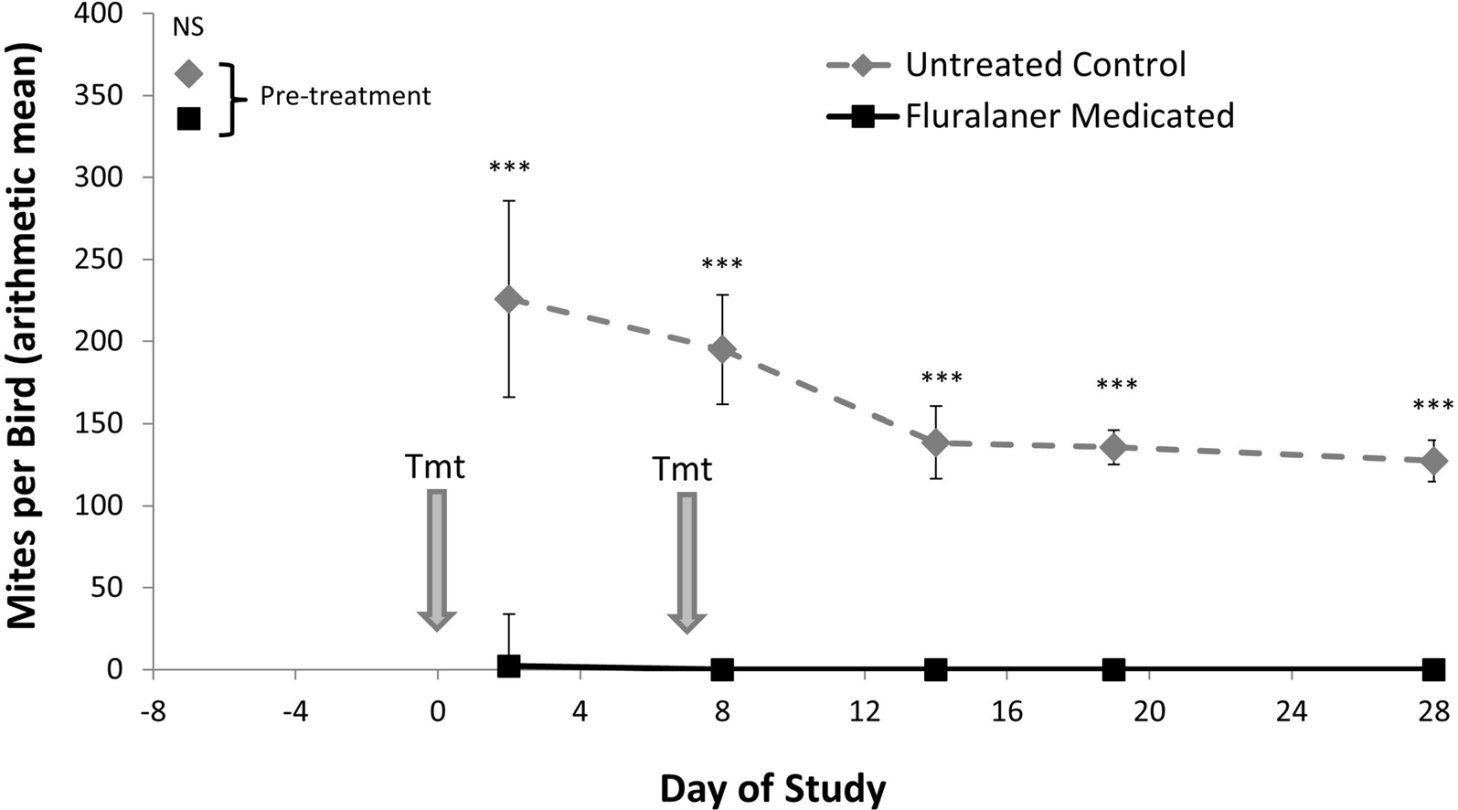
Mean number (± standard error, SE) of mites on young chickens (pullets) in the dose confirmation study. Chickens received either untreated drinking water or fluralaner administered in drinking water at 0.5 mg/kg body weight on day 0 and day 7 (treatment dates indicated with grey arrows). Significance indicated at *P* < 0.0001 (***) or not significant (NS)

A total of 128 birds were used in the dose confirmation analysis, with 64 birds in each treatment group. Chickens in the Medicated and Control treatment groups had similar pretreatment mean (SE) mite counts of 336 (31.7) and 363 (59.8) NFM per bird, respectively (*F* = 0.22; *df* = 1,30; *P* = 0.64) (Table 3, Fig. 3). From day 2 onward, mite counts differed significantly between the two treatment groups (*F* = 1006.85; *df* = 1,731; *P* < 0.0001) and across study days (*F* = 222.92; *df* = 1,731; *P* < 0.0001) when analyzed by GLMM. By day 2 post-treatment, mites were eliminated from 46 of 64 Medicated group birds while all control birds continued to have mites (Table 3). The Medicated group had a mean mite count of 2.2(0.8) NFM per bird, which was significantly lower than the 226(33.4) NFM per bird for the Control group (*F* = 34.91; *df* = 1,30; *P* < 0.0001). The few NFM remaining on Medicated group birds on day 2 post-treatment were generally observed to be recently blood-fed protonymphs. While both Medicated and Control birds experienced a significantly reduced number of mites from day −7 to day 2, the reduction in mites on Control birds (*F* = 4.38; *df* = 1,30; *P* < 0.05) was modest compared to the almost complete elimination of mites from Medicated birds over this same period (*F* = 219.64; *df* = 1,30; *P* < 0.0001). By day 2 post-treatment, control efficacy was 99% (99.6% efficacy using geometric means), exceeding the predetermined threshold for product effectiveness.

**Table 3:** Mean number (± standard error, SE) of northern fowl mites (NFM) per chicken (mite count) and control efficacy by study day and treatment group in the Dose Confirmation Study

Study Day	Treatment	Mite Count Range (min–max)	Mite Count Arithmetic Mean (± SE)	Control Efficacy Arithmetic Mean (%)	Mite Count Geometric Mean	Control Efficacy Geometric Mean (%)	F value	P value
-7	Control	135–3900	363 (59.8)	–	285	–	0.22	0.64
	Medicated	135–1675	336 (31.7)		284			
2	Control	36–1750	226 (33.4)	99	162	99.6	34.91	< 0.0001
	Medicated	0–45	2.2 (0.8)		0.6			
8	Control	39–1120	195 (22.0)	100	154	100	67.91	< 0.0001
	Medicated	0–0	0		0			
14	Control	25–460	138 (10.5)	100	115	100	103.35	< 0.0001
	Medicated	0–0	0		0			
19	Control	6–560	136 (12.5)	100	108	100	82.13	< 0.0001
	Medicated	0–0	0		0			
28	Control	2–490	127 (11.4)	100	97	100	72.31	< 0.0001
	Medicated	0–0	0		0			

Fluralaner oral solution was administered to pullet chickens (Medicated treatment group) through drinking water on study days 0 and 7

By day 8 post-treatment, 1 day after the 2nd dose of fluralaner, all 64 Medicated birds were free of NFM while all 64 Control birds remained mite-infested with a mean mite count of 195(22.0) NFM per bird (*F* = 67.91; *df* = 1,30; *P* < 0.0001). Thereafter and through the end of the study on day 28, all Medicated birds remained free of NFM whereas all Control birds continued to be mite-infested with a mean of 127–138 NFM per bird (*F* ≥ 72.31; *df* = 1,30; *P* < 0.0001). Thus, 100% control efficacy was achieved from day 8–28 in this dose confirmation study.

### Field efficacy trial

**Fig. 4:**
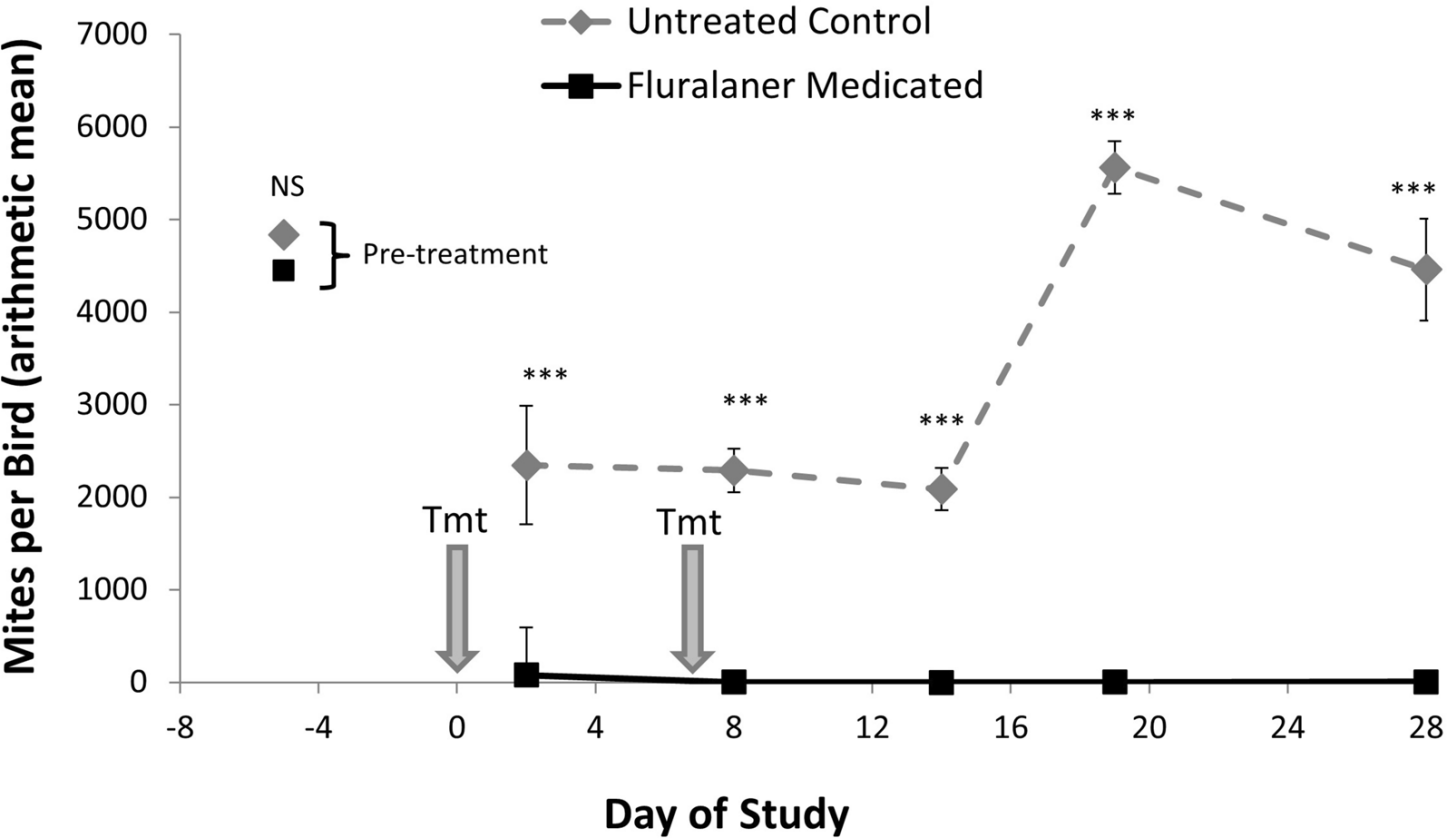
Mean number (± standard error, SE) of mites on layer chickens (~ 20 weeks old) in the field efficacy trial. Chickens received either untreated drinking water or fluralaner administered in drinking water at 0.5 mg/kg body weight on day 0 and day 7 (treatment dates indicated with grey arrows). Significance indicated at *P* < 0.0001 (***) or not significant (NS)

Of the 960 mite counts recorded from a representative sample of 80 randomly selected birds in each treatment group on each study day, nine mite counts were excluded from analyses due to discrepancies in the identification numbers associated with the birds’ leg bands. Chickens in the Medicated and Control treatment groups had similar pretreatment mean mite counts of 4450(518) and 4831(642) NFM per bird, respectively (*F* = 0.22; *df* = 1,156; *P* = 0.64 (Table 4, Fig. 4). From day 2 onward, mite counts differed significantly by treatment (*F* = 1131.96; *df* = 1,944; *P* < 0.0001) and across study days (*F* = 162.75; *df* = 5,944; *P* < 0.0001) when analyzed by GLMM. By day 2 post-treatment, the Medicated group had a significantly reduced mean mite count with 77.2(25.7) NFM per bird compared to the Control group which had a mean of 2347(238) NFM per bird (*F* = 90.75; *df* = 1,157; *P* < 0.0001). While both treatment groups experienced a significant reduction in mites from day −5 to day 2, the reduction in mite counts for Control birds was modest (*F* = 13.67; *df* = 1,152; *P* = 0.003) compared to the substantial reduction in mite count for Medicated birds over this same period (*F* = 69.31; *df* = 1,160; *P* < 0.0001). Control efficacy achieved on day 2 in this field efficacy trial was 96.7% (99.5% using geometric means), exceeding the predetermined threshold for product effectiveness.

**Table 4:** Mean number (± standard error, SE) of northern fowl mites (NFM) per chicken (mite count) and control efficacy by study day and treatment group in the Field Efficacy Trial

Study day	Treatment	Mite count range (min–max)	Mite count arithmetic mean (± SE)	Control efficacy Arithmetic mean (%)	Mite count geometric mean	Control efficacy geometric mean (%)		
							F value	P value
-5	Control	85–24,000	4831 (642)	–	2015	–	0.22	0.64
	Medicated	120–19000	4450(518)		2179			
2	Control	220–11000	2347 (238)	96.7	1545	99.5	90.75	< 0.0001
	Medicated	0–1500	77.2 (25.7)		7.7			
8	Control	125–11,000	2288 (227)	99.9	1582	> 99.9%	100.84	< 0.0001
	Medicated	0–250	3.3 (3.1)		0.1			
14	Control	75–17,000	2086 (284)	100	1376	100	55.31	< 0.0001
	Medicated	0–0	0		0			
19	Control	2–25,400	5563 (551)	> 99.9	3114	> 99.9%	100.43	< 0.0001
	Medicated	0–200	2.9 (2.6)		0.1			
28	Control	30–13700	4458 (415)	99.9	2533	> 99.9%	115.24	< 0.0001
	Medicated	0–180	5.2 (2.8)		0.4			

Fluralaner oral solution was administered to egg layer chickens (Medicated treatment group) through drinking water on study days 0 and 7

By day 8 post-treatment, 1 day after the 2nd dose of fluralaner, 75 of 80 Medicated group birds examined were free of NFM and Medicated group birds had a significantly lower mean mite count of 3.3(3.1) NFM per bird compared to the Control group birds with 2288(227) NFM per bird (*F* = 100.84; *df* = 1,158; *P* < 0.0001). Of the five Medicated birds with NFM on day 8, four birds had ≤ 7 mites and one bird had 250 mites. By day 14, all birds in the Medicated group were free of NFM while all birds in the Control group continued to be mite-infested with a mean of 2086(284) NFM per bird (*F* = 55.31; *df* = 1,156; *P* < 0.0001). On day 19, NFM were again noted on four birds in the Medicated group, including three birds with ≤ 20 mites and one bird with 200 mites. Still the Medicated group had a very low mean mite count of 2.9(2.6) NFM per bird while the Control group had a substantially higher mean of 5563(551) NFM per bird (*F* = 100.43; *df* = 1,155; *P* < 0.0001). On day 28, NFM were observed on seven Medicated birds, including five birds with ≤ 35 mites, one bird with 135 mites and one bird with 180 mites, with a mean mite count for Medicated and Control birds of 5.2(2.8) and 4458(415) NFM per bird, respectively (*F* = 115.24; *df* = 1158; *P* < 0.0001). Control efficacy exceeded 99.9% from day 8 to day 28 in this field efficacy trial.

**Fig. 5:**
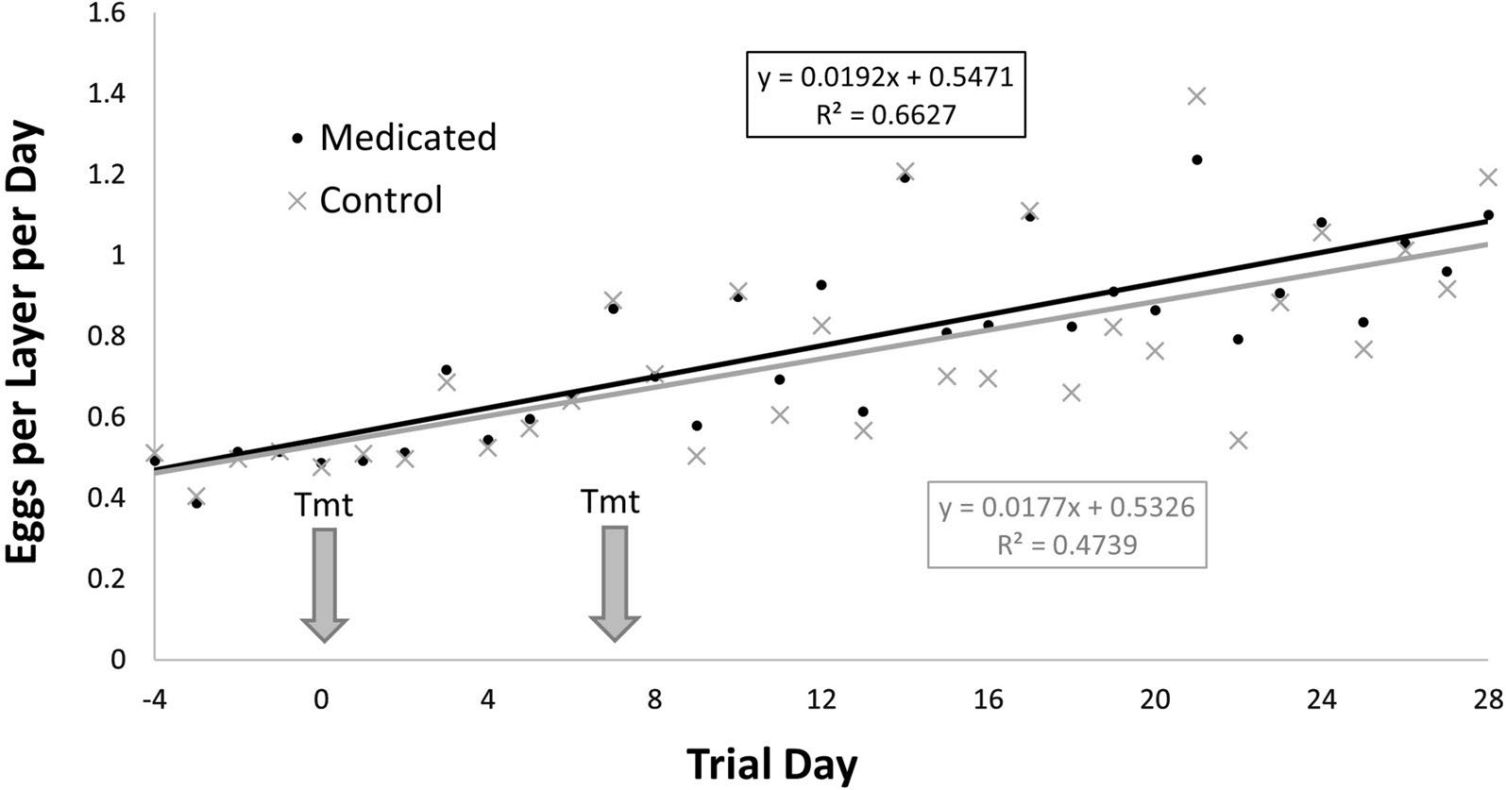
Egg production (eggs/bird/day) for Medicated and Control treatment groups. Egg production per treatment group (400 layers in each group) was recorded daily from day −4 through day 28 post-treatment. Fluralaner was administered to Medicated group birds on days 0 and 7 as indicated by arrows

Egg production was similar among birds in the two treatment groups prior to first administration of fluralaner (day −4 to −1) (*t* = 0.57; *df* = 1,3; *P* = 0.6) with birds in the Control group producing 0.005 eggs/bird/day more than birds in the Medicated group. However, after administration of fluralaner, daily egg production was significantly greater for birds in the Medicated treatment group than for birds in the Control group (*t* = 2.64; *df* = 1,27; *P* = 0.014) (Fig. 5), with the Medicated group birds producing an average of 0.04 eggs/bird/day more than untreated control birds during the 28-day post-treatment period.

## Discussion

Control of NFM on poultry has traditionally relied on acaricides applied directly to the vent region of birds by high-pressure spray directed from beneath the bird to penetrate the feather layer and treat mites at the host skin surface [2]. However, the majority of acaricide sprays currently available in the USA for control of NFM contain a pyrethroid as the active ingredient [28], and practical use of these products is limited by NFM resistance to pyrethroids [19, 29]. Although a few acaricide sprays containing other active ingredients (spinosyns or organophosphates) remain available for control of NFM in the USA [28], the recent shift in poultry housing design to enriched cage or cage-free housing [4] has made it difficult to apply these products to the vent region of birds in commercial poultry production. While sulfur dust, kaolin clay, or diatomaceous earth (DE) applied directly to birds or as an amendment to dustboxes or dustbags can reduce NFM on poultry [30–32], birds require both access to and use of dustboxes for these products to be effective. Given the challenges of acaricide resistance and changing animal housing, there is substantial need for new methods to control NFM in commercial poultry facilities.

In both the dose confirmation study and field efficacy study presented here, mite counts on treated birds were rapidly reduced within 2 days post-treatment. Mites were eliminated from Medicated birds by day 8 in the dose confirmation study and by day 14 in the field efficacy trial. Birds in both studies exceeded the predetermined product efficacy threshold of > 90% from day 2 through day 28 when the study ended.

These studies were conducted under field conditions, with the field efficacy study conducted on a commercial layer operation following commercial practices and procedures for bird management. Such field studies add real-world complexity to drug studies and are required for drug approval in the USA to ensure efficacy of medication under conditions of anticipated use. While animal studies conducted in single-animal isolators can provide greater control over the concentration and timing of medication to individual chickens as well as control environmental variables, potentially also reducing variation in outcome data, such a study would be insufficient to demonstrate product efficacy in a commercial setting.

A limitation of the Kruskal–Wallis test is that its conservative nature can reduce sensitivity to small or subtle effects. However, given the highly skewed and zero-inflated distribution of mite counts in these studies, the use of a nonparametric approach was appropriate. Moreover, the very large and consistent treatment effects observed in these studies provide strong evidence that fluralaner medication administered to chickens in drinking water exerts a substantial and reproducible effect on NFM infestation of treated birds.

This study supports previous research demonstrating effective control of NFM when fluralaner was administered by oral gavage to laying hens as two single doses of 0.25–1.0 mg/kg BW applied at 7 days apart [16], though in contrast to the current studies, complete elimination of mites from fluralaner-treated birds was never achieved in this prior study, as treated and control birds were co-mingled throughout the study period.

The rapid control of NFM after administration of fluralaner in the current studies was similar to previous studies with fluralaner administered at the same dose for control of the poultry red mite (*D. gallinae*) in Europe [21, 22], even though the host association habits of these two blood-feeding mites vary considerably [5]. In these previous studies, > 96% mite control efficacy was achieved by day 3 when fluralaner was administered to birds of various ages and breeds in varied commercial production farms [22], and 100% mite control efficacy was achieved by day 7 when fluralaner was administered to birds housed in an aviary [21].

In the dose confirmation study, the few mites observed alive on Medicated birds on day 2 post-treatment were mostly protonymphs containing bright red host blood suggesting a very recent bloodmeal. These blood-engorged protonymphs were very likely in the egg stage at the time of the first administration of fluralaner on day 0 and thus were not exposed to fluralaner until completing their nonfeeding immature life stages and subsequently reaching the blood-feeding protonymph stage. Because mites are killed within 4 h of consuming blood containing fluralaner at the dose used in this study [18], it is likely these mites had consumed host blood within just a few hours before mite counts were performed on day 2.

Fluralaner administered to chickens in drinking water at the 0.5 mg/kg BW dose used in this study provided 100% mortality of poultry red mite for up to 5 days with a second administered dose 7 days after the first, extending the 100% mortality period another 5 days (to 12 days after the first treatment) [17]. If the protective period for NFM is similar to that reported for poultry red mite, reinfestation of treated birds by NFM might be expected after the 5-day protective period following the last administration of fluralaner. Indeed, in the field efficacy study where study birds were housed in open-sided poultry houses allowing access by wild birds and thus continuous opportunity for NFM reinfestation, a small number of NFM were observed on a few treated birds at days 19 and 28 following the earlier elimination of NFM by day 14.

In contrast to the field efficacy study, NFM were eliminated throughout the entire 28-day study period in the dose confirmation study. The lack of NFM reinfestation in the dose confirmation study can be attributed to the great care of the research team to prevent accidental transfer of mites from untreated to treated birds by personnel, equipment, or other means. Additionally, the exclusion of wild birds from the poultry house used in this study and the separation of cages holding untreated and treated birds by 1 m of bare earth seems to have been sufficient to prevent movement of NFM from untreated to treated chickens for at least 21 days following the last administration of fluralaner. In a commercial poultry production setting, strict protocols to prevent accidental mite transfer by personnel or equipment along with housing modifications to separate chickens from wild birds can therefore extend the mite-free period following fluralaner treatment. In European studies, control of poultry red mite was achieved for two or more months [22] or for up to 6 weeks in aviary and free-range farms [21], until treated poultry houses were reinfested due to inadequate separation of fluralaner-treated and untreated chickens.

NFM can reduce egg production in white Leghorn hens [9, 33], which along with other negative impacts including reduced egg weight and reduced feed conversion can reduce profits for poultry producers by $0.01–0.014 per hen per week [9]. After fluralaner administration, egg production was greater for Medicated group birds than for Control group birds, resulting in an additional 1.14 eggs/layer over the 28-day trial for birds in the Medicated group relative to birds in the Control group. Treatment of layers with fluralaner to control the related poultry red mite was similarly shown to increase egg production in treated birds [22, 34] or to decrease the rate of decline in egg production over time relative to published standards for birds of the same age and strain [21]. However, the increased egg production by medicated birds reported in the current study should be taken with caution because the current study was not designed specifically to assess changes in egg production, and the impact of other potentially influencing factors, such as the assignment of each treatment group to a single cage row, cannot be fully assessed. The increase in egg production demonstrated in the current study following fluralaner administration should be confirmed in future studies.

Elimination of NFM from layers results in reduced bird preening and fewer skin lesions relative to mite-infested birds [35]. Similarly, elimination of the nighttime feeding poultry red mite following fluralaner administration to layers resulted in a reduction in nighttime bird activities (e.g., head shaking and preening) and an increase in the percentage of hens resting at night [21]. Although bird behaviors were not observed in the current study, the substantial reduction in mite numbers, including mite elimination from many treated birds, might be expected to improve bird health and welfare [35, 36]. Additional studies would confirm egg production or other economic benefits associated with fluralaner treatment against NFM.

## Conclusions

Treatment of egg layer chickens with fluralaner oral solution administered in drinking water at two single doses of 0.5 mg/kg chicken BW 7 days apart is effective for control of NFM. On day 2 post-treatment, 99% control efficacy (> 99% for geometric mean) was achieved in the dose confirmation study and > 96% (> 99% for geometric mean) control efficacy was achieved in the field efficacy trial, demonstrating rapid control. Control efficacy in both studies exceeded 99% from day 8 through 28, and there were no adverse health impacts observed in birds treated with fluralaner. Furthermore, these studies demonstrated elimination of mites from medicated birds as early as day 8, thus indicating the potential for fluralaner to deliver both rapid control and probable elimination of NFM from a flock, at least until mites are reintroduced to the flock from outside sources.

## Data Availability

The datasets used and/or analyzed during the current study are curated and available to the public through Dryad at 10.5061/dryad.9cnp5hqzs

## Abbreviations

AE: Adverse event
BW: Body weight
LC: Liquid chromatography
LOD: Limit of detection
LOQ: Limit of quantitation
NFM: Northern fowl mite
UCR: University of California at Riverside
UPLC: Ultra performance liquid chromatography
UV: Ultraviolet
